# Experimental and theoretical investigations of benzoic acid derivatives as corrosion inhibitors for AISI 316 stainless steel in hydrochloric acid medium: DFT and Monte Carlo simulations on the Fe (110) surface†

**DOI:** 10.1039/d0ra06742c

**Published:** 2020-11-12

**Authors:** Mustapha Alahiane, Rachid Oukhrib, Youssef Ait Albrimi, Hicham Abou Oualid, Hassan Bourzi, Rachid Ait Akbour, Ali Assabbane, Ayssar Nahlé, Mohamed Hamdani

**Affiliations:** Ibn Zohr University, Science Faculty, Chemical Department Agadir Morocco; Applied Chemistry-Physics Team, Faculty of Sciences, University of Ibn Zohr Agadir Morocco; Laboratory of Biotechnology, Materials and Environment, Faculty of Sciences, Ibn Zohr University Agadir Morocco H.abououalid@gmail.com; Green Energy Park, IRESEN, UM6P Benguerir Morocco; Department of Chemistry, University of Sharjah, College of Sciences PO Box 27272 Sharjah United Arab Emirates

## Abstract

The inhibition efficiency of benzoic acid (C1), *para*-hydroxybenzoic acid (C2), and 3,4-dihydroxybenzoic acid (C3) towards enhancing the corrosion resistance of austenitic AISI 316 stainless steel (SS) has been evaluated in 0.5 M HCl using weight loss (WL), open circuit potential (OCP), potentiodynamic polarization method, electrochemical impedance spectroscopy (EIS), and scanning electron microscopy (SEM) analysis. The results obtained from the different experimental techniques were consistent and showed that the inhibition efficiency of these inhibitors increased with the increase in concentration in this order C3 > C2 > C1. In addition, the results of the weight loss measurements showed that these inhibitors followed the Villamil isotherm. Quantum chemical calculations and Monte Carlo simulations have also been used for further insight into the adsorption mechanism of the inhibitor molecules on Fe (110). The quantum chemical parameters have been calculated by density functional theory (DFT) at the B3LYP level of theory with 6-31G+(2d,p) and 6-31G++(2d,p) basis sets in gas and aqueous phase. Parameters such as the lowest unoccupied (*E*_LUMO_) and highest occupied (*E*_HOMO_) molecular orbital energies, energy gap (Δ*E*), chemical hardness (*η*), softness (*σ*), electronegativity (*χ*), electrophilicity (*ω*), and nucleophilicity (*ε*) were calculated and showed the anti-corrosive properties of C1, C2 and C3. Moreover, theoretical vibrational spectra were calculated to exhibit the functional hydroxyl groups (OH) in the studied compounds. In agreement with the experimental data, the theoretical results showed that the order of inhibition efficiency was C3 > C2 > C1.

## Abstract 2

The corrosion inhibition efficiencies of benzoic acid (C1), para-hydroxybenzoic acid (C2), and 3,4-dihydroxybenzoic acid (C3) have been evaluated in 0.5 M HCl toward protecting AISI 316 stainless steel (SS).

## Graphical abstract

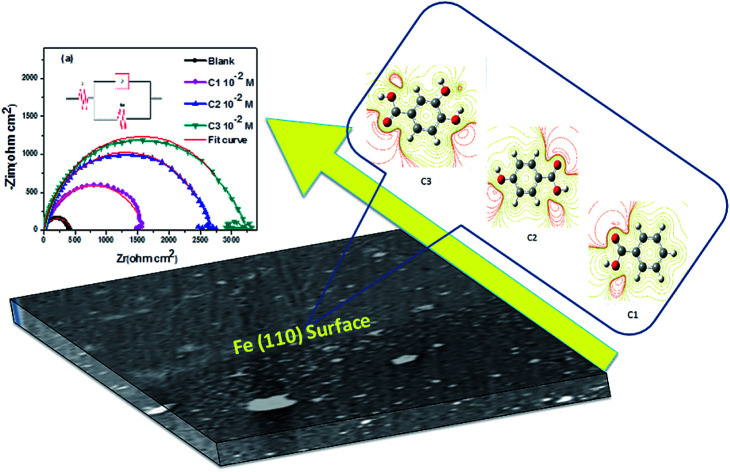

## 1. Introduction

The study of corrosion inhibitors has become an important industrial and academic topic due to their economic implications. Therefore, researchers have being addressing this subject in many ways, especially to present fundamental aspects of metal corrosion phenomena and corrosion inhibition across scientific research.^1–4^ Most research projects deal with the electrochemical principles and chemical aspects of corrosion inhibition, such as the stability of metals and their alloys, different corrosive media, quantum chemical aspects, and also with the various surface analysis techniques that are used in industry and the academic field to deepen the diagnosis and studies for corrosion inhibition.^5^ In the same context, the understanding of stainless steel corrosion is also considered an essential topic for academic and industrial fields^6^ due to its wide applications in acidic industries such as petroleum platforms, oil well acidification, chemical handling, and water treatment, as well as in surface treatment plants for descaling, pickling, and rust removal.^7–9^ The application of stainless steel in aggressive environments is subject to corrosion, which generates huge financial losses in industrial processes.^10–13^ Therefore, it is essential to find solutions to the corrosion of materials used in aggressive environments.

Austenitic stainless steels are the most widespread materials due to their high corrosion resistance and relatively low cost. They have a wide variety of applications in seawater desalination, food and beverage industry, and other applications. It is well known that a passive protective film is rapidly formed on the surface of steels. Their high strength is related to its excellent corrosion resistance. Yet this layer is damaged in an acidic environment due to the presence of certain aggressive ions such as chlorides.^14^ The corrosion resistance of passive stainless steels is limited by the local rupture and nucleation of pits on their surface. To remedy this corrosion problem, non-toxic corrosion inhibitors with good inhibition efficiency are available. Our study is focusing on benzoic acid derivatives. The prevention of the corrosion has been the subject of great interest aiming to protect the equipment. The use of organic inhibitors is one of the usual ways to prevent the corrosion of metals, particularly in acidic media.^15,16^ A variety of organic compounds containing heteroatoms (N, O, S) possess lone-pair electrons or heterocyclic compounds having polar groups and π electrons and lead to electron transfer to the metallic surface and consequently are used successfully to inhibit the corrosion of metal in various aggressive electrolytes through their adsorption on the metal surface.^17–20^ These substances react based on their affinities to be firmly adsorbed on the surface of metals through the electron density of the functional groups as active sites on the exposed surface, thereby preventing the corrosive action in the acidic medium according to several studies.^21–23^ In order to react across electrons density of the donor atoms or actives sites and linked to possible steric effects and electronic effects, the adsorption of the inhibitor molecule depends on the physicochemical properties. In addition, the adsorption also depends on the nature of the metal surface, the chemical composition of the solution, the electrochemical potential at the metal-solution interface, the temperature, and the pH of the aggressive medium.^24–28^ Previous studies have shown the importance of a variety of organic compounds such as benzoic and salicylic acids which were used as corrosion inhibitors for steel in acidic media. Both compounds have partially inhibited the corrosion of steel, but the former was more efficient than the later at equal concentrations. This result was explained by the ability of the benzoic acid to form stable dimeric structures, which protect more the steel under the prevailing conditions.^29–33^

Abdallah *et al.*^34^ reported on the corrosion inhibition of carbon steel in hydrochloric acid solution using some phenolic compounds such as *o*-aminophenol (1), catechol (2), salicylaldehyde (3), and salicylic acid (4), and they concluded that the use of the same inhibitors concentrations decreased the inhibition efficiency of the investigated compounds in the sequence from 1 to 4, and this was supported by quantum chemical parameters. The same authors^34^ have also studied the synergic effect of KI, KSCN, and KBr. The corrosion rate process decreased in the following order: iodide > thiocyanate > bromide. The strong chemisorption of iodide, thiocyanate, and bromide ions on the metal surface is responsible for the synergistic effect of these anions in combination with the cation of the inhibitor. Furthermore, the synergic effect of KI with some heterocyclic inhibitors on the protection of AISI 304 stainless steel in 1.0 M HCl was investigated.^35^ Other organic acids (*i.e.*, phthalic acid, salicylic acid, benzoic acid, *o*-aminobenzoic acid, and oxalic acid) were used to hint pitting corrosion of C-steel in a 0.01 M NaOH solution containing Cl^−^ ions.^36^

In this our study, the efficiency of three carboxylic acids, namely benzoic acid (C1), 4-hydroxybenzoic acid (C2), and 3,4-dihydroxybenzoic acid (C3) against the corrosion of 316 SS in 0.5 M HCl has been determined and compared using experimental and theoretical methods (DFT study).^37^ These three compounds that are commercially available, less expensive and eco-friendly, show good effectiveness against corrosion of 316 SS in the studied acid medium. The inhibitor C3 (3,4-dihydroxybenzoic acid) acts as a better inhibitor compared to the inhibitors C2 (*para*-hydroxybenzoic acid) and C1 (benzoic acid) at the same concentrations. The obtained experimental results are in good agreement with the theoretical results determined in this study by density functional theory (DFT) at the B3LYP level of theory with 6-31G+(2d,p) and 6-31G++(2d,p) basis sets.

## 2. Experimental

### 2.1. Materials and methods

#### 2.1.1. Chemicals

Benzoic acid (C1), 4-hydroxybenzoic acid (C2), and 3,4-dihydroxybenzoic acid (C3) were purchased from Sigma-Aldrich and used as received. 0.5 mol L^−1^ HCl solutions without inhibitor (blank), and with inhibitors C1, C2, and C3, with concentrations ranging from 1.0 × 10^−6^ M to 1.0 × 10^−2^ M were used. Plates of the SS AISI 316 (Good fellow, UK) were used to carry out this work. The chemical composition of the SS was 17 wt% Cr, 12 wt% Ni, 0.18 wt% Mn, 2.05 wt% Mo, 0.08 wt% C, 1.5 wt% Mn, 0.03 wt% P, 0.60 wt% Si, and Fe in balance. Samples (1 cm × 1 cm × 0.1 cm) were cut from the SS sheet. Each side was ground with 320 and 1200 grid emery papers, degreased ultrasonically with ethanol, before drying in the oven.

#### 2.1.2. Electrolyte solution

The HCl solutions used in this work were prepared by dilution of a concentrated HCl solution of density *d* = 1.18 and percentage by mass is between 35% and 38% using deionized water. For the preparation of 1 L solutions containing 1.0 × 10^−2^ M inhibitor(s), the desired quantity of each inhibitor (1.22 g C1; 1.38 g C2; 1.54 g C3) was added to 0.5 M HCl. Inhibitors concentrations ranging from 1.0 × 10^−6^ M to 1.0 × 10^−2^ M were prepared in 0.5 M HCl.

#### 2.1.3. Electrochemical measurements

Electrochemical investigations were performed in a three electrodes glass cell, *i.e.* working electrode (WE) (SS), saturated calomel electrode (SCE) (0.240 V *vs.* SHE), and a platinum plate of 3 cm^2^ surface area as a counter electrode (CE). The KCl-agar-agar salt bridge was used to minimize the ohmic resistance between the WE and the RE. 100 mL of aerated and unstirred 0.5 M HCl with and without inhibitors was used in each experiment. The work was carried out using VoltaLab PGZ 100 (Radiometer-Analytical) potentiostat controlled by a computer provided with an electrochemical software. Prior to potentiodynamic polarization, the WE open-circuit potential (OCP) was controlled for at least 30 min. The polarization curves were performed by sweeping the electrode potential in the interval from −1.0 V to +1.0 V *vs.* SCE using a scan rate, *v*, of 1 mV s^−1^. In order to calculate the corrosion current densities, the cathodic branch of Tafel curve was extrapolated to the corrosion potential, *E*_corr_. The polarization resistance, *R*_p_, was also determined in the *E*_corr_ interval of ±10 mV. The electrochemical impedance spectroscopy, (EIS), was carried out after OCP measurement. The OCP steady state was reached at less than 30 min of immersion in the solution. EIS measurements were carried out at OCP within the frequency range of 1.0 × 10^5^ to 1.0 × 10^−2^ Hz using alternating current voltage amplitude of 10 mV peak-to-peak voltage excitation. The experiments were repeated three times to ensure the reproducibility and the results were given in Nyquist form.

#### 2.1.4. Gravimetric method

Weight loss (WL) measurements were carried out using pre-cleaned specimens hanged in 100 mL of uninhibited and inhibited 0.5 M HCl at a constant temperature in a controlled thermal chamber. WL measurement was performed by weighting the coupons after regular immersion time and the SS weight loss was considered as the mean weight loss of triplicate specimens under the same conditions. The degree of metal surface coverage (*θ*), and the inhibition efficiency, IE (%), of the investigated compounds were calculated respectively using eqn (1) and (2).1
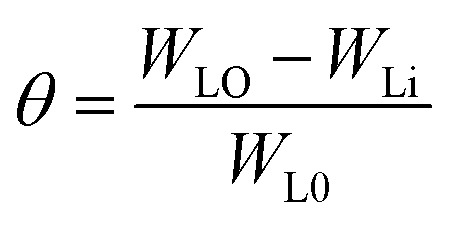

2 IE% = *θ* × 100

where *W*_L0_ and *W*_Li_ are the weight-loss per surface area for uninhibited and inhibited solutions at a given concentration, respectively.

#### 2.1.5. Scanning electron microscopy

Surface morphologies analysis of SS specimens was performed using a high-resolution scanning electron microscope (SEM) equipped by FEI, Quanta 200-ESEM, at the accelerating voltage of 20 kV. The sample morphologies were carried out before and after 48 hours of immersion in 0.5 M HCl in the absence and in the presence of C1, C2, and C3 inhibitors. After immersion, and before the SEM analysis, each specimen was washed with ultra-distilled water and dried at atmospheric air.

### 2.2. Quantum chemical studies

Using Gaussian 03 W program package, the DFT calculations were performed on the studied three benzoic acid derivatives (C1), (C2), and (C3) in gas and aqueous phases as well. All molecules were geometrically optimized using DFT/B3LYP method associated with 6-31G++(2d,p) basis set.^38–42^ The optimization process was confirmed by the absence of imaginary vibration frequencies. Afterward, several relevant molecular electronic structure parameters (global and locale indicators) were calculated. These included lowest unoccupied (*E*_LUMO_) and highest occupied (*E*_HOMO_) molecular orbital energy, as well as gap energy (Δ*E*, eqn (3)), electronegativity (χ, eqn (4)), hardness (*η*, eqn (5)), fraction of electrons transferred (Δ*N*, eqn (6)), and dipole moment (*μ*). Furthermore, the frontier molecular orbitals (*i.e.*, HOMO and LUMO) repartitions and 2D electrostatic potential plots of each benzoic acid derivative were calculated and illustrated.

3 Δ*E* = *E*_LUMO_ − *E*_HOMO_

4
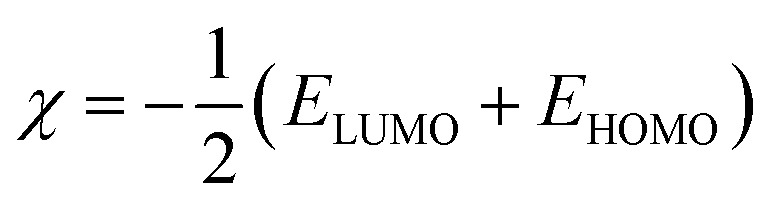
5
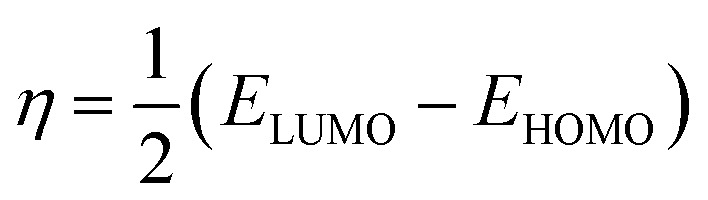
6
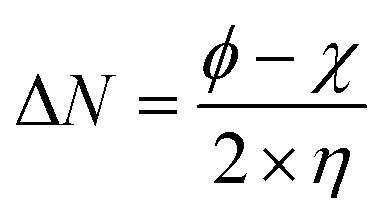
where *ϕ* is the work function of the metal surface (*ϕ*_Fe_ = 4.81 eV).^43,44^

### 2.3. Monte Carlo simulation

The interaction between the investigated three benzoic acid derivatives compounds and the selected metal surface Fe (110) was studied using Monte Carlo simulations allied to the simulated annealing (SA) algorithm in the gas phase.^45–47^ To predict the more adapted metallic surfaces for the simulations process, Bravais–Friedel–Donnay–Harker (BFDH) method was used.^48^ Five layers and a vacuum region of 60 Å was used to model the slab of each studied metal in this current work. The van der Waals and the electrostatic interactions were calculated by the atom-based method and Ewald summation method, respectively. Materials Studio 2017 software was utilized to perform these calculations with COMPASS force field. The adsorption energy (*E*_ads_) of the three benzoic acid derivatives on the metal surface was calculated according to the following expression.^49,50^

7 *E*_ads_ = *E*_T_ − (*E*_surf_ + *E*_ads_)

where, *E*_T_ denotes the total energy of the whole system, *E*_surf_ is the energy of metal surface, and *E*_ads_ is the energy of one of the three derivative compounds adsorbed on the metal surface (Fig. A1).†

## 3. Results and discussion

### 3.1. Electrochemical studies

#### 3.1.1. Open circuit potential measurement

**Fig. 1:**
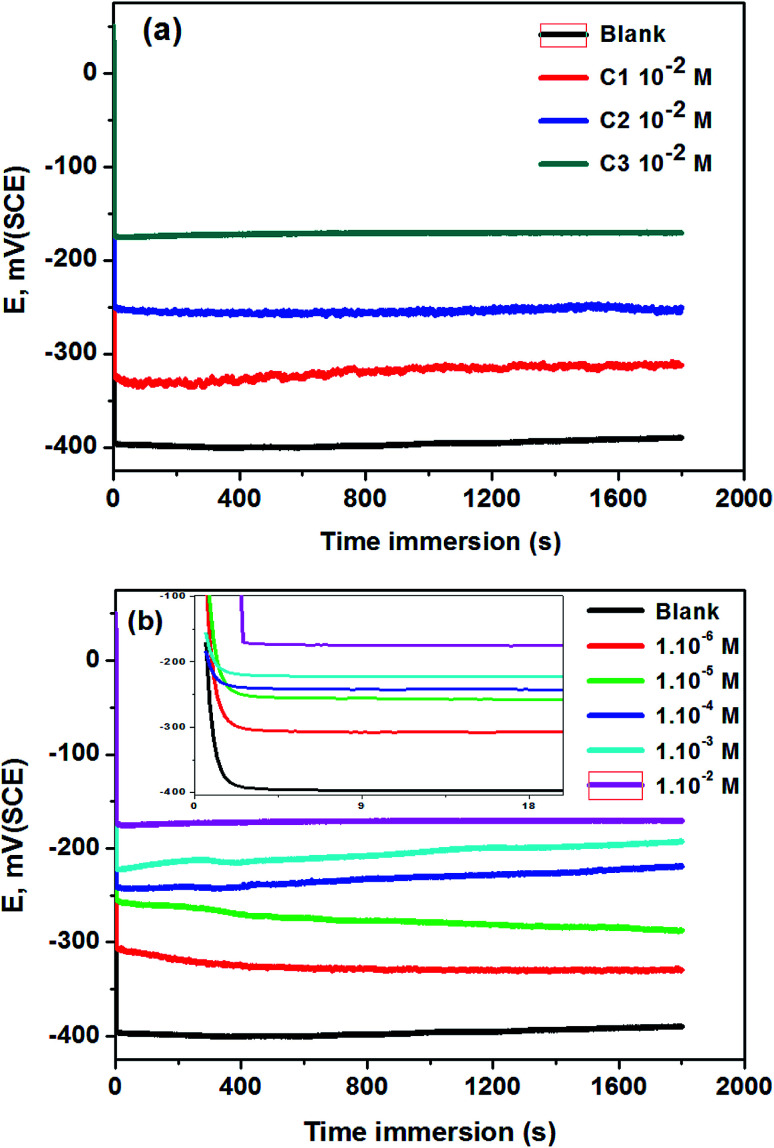
OCP curves of 316 SS in 0.5 M HCl; (a) without and with 1.0 × 10^−2^ M of C1, C2, and C3; (b) at different concentrations of C3 at 291 K (the insert represents the shape of the curve at the first 10 seconds).

The open-circuit potential (OCP) measurements of 316 SS electrodes in 0.5 M HCl without and with C1, C2, and C3 inhibitors at 1.0 × 10^−2^ M are shown in Fig. 1a. The OCP stabilized at −400 mV *vs.* SCE in the uninhibited solution up to 30 minutes. In the case of inhibited solutions, OCP shifted anodically towards noble potential and stabilized at −325, −250, and −175 mV *vs.* SCE in 0.5 M HCl containing 1.0 × 10^−2^ M of C1, C2, and C3 respectively. The difference of OCP values of SS electrodes using the three inhibitors confirms the difference of surface protection of the inhibitors against the steel corrosion. The anodic shift of the OCP testifies the better protection of steel in 0.5 M HCl containing 1.0 × 10^−2^ M of C3 than those containing C1 and C2. The effect of the concentration of C3 in 0.5 M HCl on the OCP electrodes is shown in Fig. 1b. The OCP values remain generally constant after ten seconds and increased with the increase of C3 concentrations (Fig. 1b insert). This observation confirms that the inhibition efficiency of C3 against SS corrosion depends on the concentration of the inhibitor.

#### 3.1.2. Potentiodynamic polarization studies

**Fig. 2:**
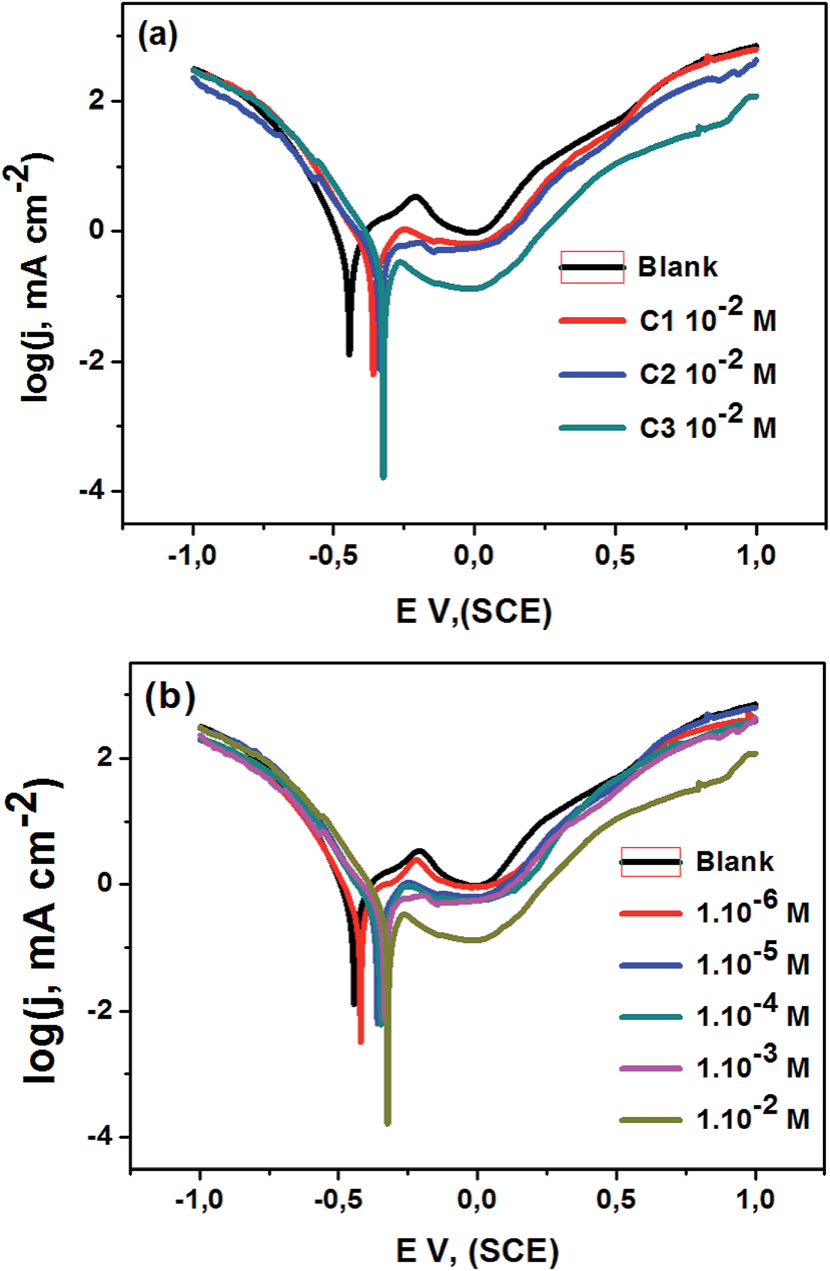
Polarization curves of steel 316 in 0.5 M HCl medium; (a) with and without 1.0 × 10^2^ M of the C1, C2, and C3; (b) with different concentrations of C3; *T* = 291 K; *v* = 1 mV s^−1^.

**Fig. 3:**
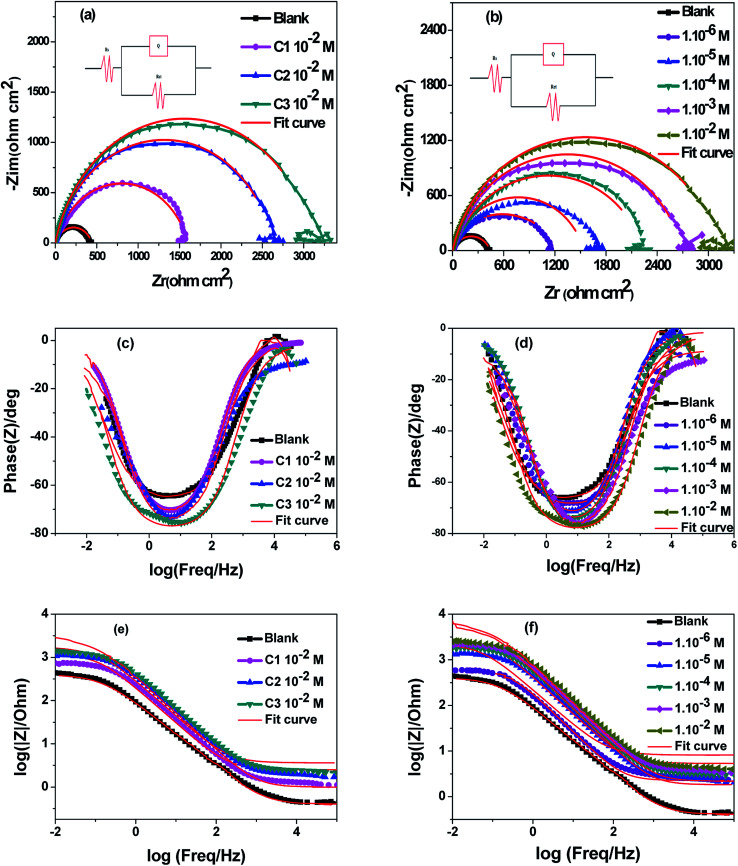
Fitted Nyquist plots and Bode diagrams of 316 steel in 0.5 M HCl; (a), (c) and (e) without and with 1.0 × 10^−2^ M of C1, C2 and C3; (b), (d) and (f) without and with various concentrations of C3, at *T* = 291 K.

The 316 stainless steel corrosion resistance was studied using potentiodynamic technique in 0.5 M HCl solution and in the presence of C1, C2, and C3 inhibitors. Fig. 2a and b show log(*I*) − *E* curves for SS in uninhibited and inhibited solutions at a scan rate of 1 mV s^−1^. The three compounds inhibit partially the corrosion of the steel. The anodic part of the potentiodynamic curve obtained for the SS in the blank solution exhibits active process, which disappeared in the presence of the inhibitors. The corrosion potential, *E*_corr_, increased while the current densities decreased in the inhibited solutions. C3 acts as a better inhibitor compared to C1 and C2 compounds at equal concentrations. It seems that C3 protects the SS better than C2 and C1 in accordance with the obtained results from OCP measurements. The current densities decreased with increasing the concentration of C3 inhibitor in the range of 1.0 × 10^−6^ to 1.0 × 10^−2^ M (Fig. 3b). The inhibition efficiency was calculated using the corrosion current densities and according to the following expression eqn (8).8
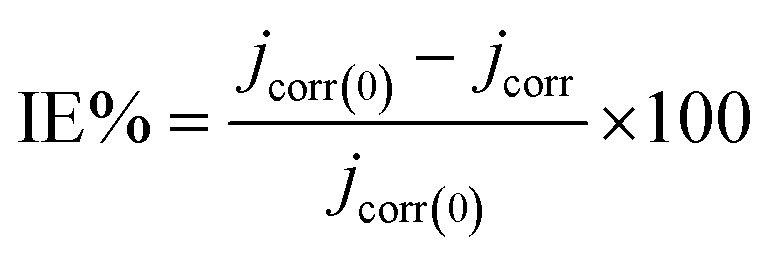
where *j*_corr(0)_ and *j*_corr_ are the corrosion current densities of uninhibited and inhibited solutions.

Various corrosion parameters *i.e.*, corrosion current density, *J*_corr_, corrosion potential, *E*_corr_, cathodic Tafel slope, *β*_c_, and inhibition efficiency, IE, deduced from these curves are encapsulated in Table 1. The inhibition efficiency was calculated using corrosion current densities. Cathodic Tafel slope, for hydrogen evolution, decreased in the inhibited solution, which refers to the mixed type inhibition. The cathodic branch of the polarization curves in uninhibited and inhibited solutions presents the same shape, indicating that the mechanism of H_2_ evolution reaction does not change.

**Table 1:** Electrochemical parameters of 316 stainless steel in 0.5 M HCl with and without 1.0 × 10^−2^ M of C1, C2, and various concentrations of C3; at *T* = 291 K

Inhibitor concentration in 0.5 M HCl (M)		−*E*_corr_ (mV/SCE)	*j*_corr_ (μA cm^−2^)	−*β*_c_ (mV dec^−1^)	IE^a^ (%)
Blank		452	749.9	168.8	—
C1	1.0 × 10^−2^ M	363	204.7	159.7	72.7
C2	1.0 × 10^−2^ M	335	119.6	111.1	84.1
C3	1.0 × 10^−6^ M	419	275.96	163.0	63.2
	1.0 × 10^−5^ M	367	188.0	159.0	74.9
	1.0 × 10^−4^ M	357	136.0	135.0	81.8
	1.0 × 10^−3^ M	343	125.0	120.0	83.3
	1.0 × 10^−2^ M	324	88.0	113.0	88.2

a Inhibition efficiency IE, calculated using corrosion current densities.

#### 3.1.3. Impedance measurements

The electrode/electrolyte interface of AISI 316 austenitic SS electrode has been studied using electrochemical impedance spectroscopy (EIS) technique to get information about the interfacial electrode processes. The impedance spectra of SS electrode were measured in uninhibited 0.5 M HCl and inhibited with C1, C2, and C3. The Nyquist plots and fitted Bode diagrams are updated after 30 minutes of immersion in uninhibited and inhibited solutions, the Fig. 3 displayed the obtained data.

From the fitted Nyquist plots the inhibition performances of C1, C2, and C3 were compared at the same concentration are showed in (Fig. 3a and b). All the curves showed at high frequencies a semicircle capacitive loop shape. The charge transfer resistance (*R*_t_) values are calculated from the diameter of the semicircle. This parameter increased in the following sequence; blank solution, blank solution containing C1, blank solution containing C2, and blank solution containing C3. The corrosion protection performance evolved in the same way; the protection performance of C3 is better than that of both C1 and C2. Subsequently, the behavior of the electrode in the aggressive medium with various concentrations of C3 was also studied (Fig. 3b). Indeed, the diameter of the semicircles observed in the inhibited solution is superior to those obtained in blank solution. It also increases with the progressive increase of inhibitor content in the solution, which may be referred to the enhancement of the metal surface protection by the surface coverage of the inhibitor. In this sense, the close inspection of Fig. 3b showed that *R*_p_ or the corrosion resistance increased by increasing the concentration of C3 in the medium. Obtaining a semicircle shape with one loop refers to the dissolution of the SS electrode with a single charge transfer process. The obtained Nyquist plots are in a semicircle shape, which is due to the frequency dispersion of interfacial impedance, the surface roughness, the chemical heterogeneity of surface, and the adsorption–desorption process of inhibitive molecules on steel surface.^51–53^

The Bode (Fig. 3c and d) phase angle plots show a single peak at intermediate frequencies indicating the presence of one time constant. Moreover, the Bode plot obtained in presence of our inhibitors displayed only one phase maximum, indicating only one relaxation process. Thus, charge transfer process could be taken place at the metal-electrolyte interface. It is also observed from Bode plots (Fig. 3e and f) that a linear relationship between log/*Z*/*vs.* log(*f*) was showed at the intermittence frequency region explaining the phase angle which is less than −90° and the slope value close to −1.^54^ Concerning the Bode phase plots, the increase in phase angle with increasing concentrations of benzoic acid derivatives indicates a superior inhibitory behavior due to molecules adsorbed on the surface of the steel at higher concentrations. In this, a protective film is formed on the surface of the steel.^55^ The single-phase peak that can be observed on the Bode phase graphs indicates that there is a single time constant for binding to the electrical double layer.^56^ The increase of the phase angle with the increase of the concentration of the tested derivatives can be attributed to the decrease of the capacitive behavior at the steel surface due to the decrease of the dissolution rate of the steel.^57^

The impedance parameters for corrosion of SS electrodes in uninhibited and inhibited acid solutions, at 291 K are encapsulated in Table 2. The ohmic resistance of the solutions is in the interval of 1.4–3.3 Ω cm^2^. The charge transfer resistance of SS interface in C3 inhibited solution goes from 1122 to 3281 Ω cm^2^ when C3 inhibitor concentrations increased from 1.0 × 10^−6^ to 1.0 × 10^−2^ M. The inhibition efficiency, IE_ct_, is expressed by the formula:
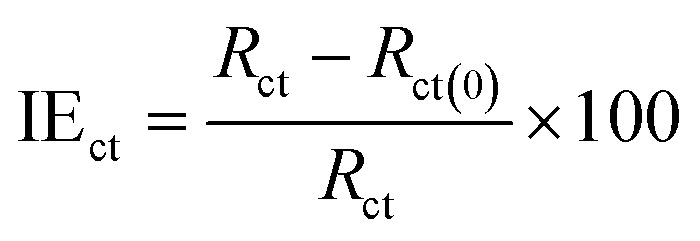
9where IE_ct_ is the inhibition efficiency, *R*_ct_ and *R*_ct(0)_ denoted the charge-transfer resistance values for uninhibited and inhibited solutions, respectively. The inhibition efficiencies are in the same trend and range of those previously obtained by other techniques.

**Table 2:** EIS parameters of 316 steel in 0.5 M HCl solution containing 1.0 × 10^−2^ M of C1, C2, and various concentrations of C3, at *T* = 291 K

Concentration of inhibitor in 0.5 M HCl (M)		*R*_s_ (Ω cm^2^)	*R*_ct_ (Ω cm^2^)	IE_ct_ (%)
Blank		2.5	424	—
C1	1.0 × 10^−2^	2.6	1519	72.1
C2	1.0 × 10^−2^	3.3	2687	84.2
C3	1.0 × 10^−6^	1.7	1122	62.2
	1.0 × 10^−5^	1.4	1686	74.8
	1.0 × 10^−4^	3.2	2243	81.1
	1.0 × 10^−3^	2.1	2719	84.4
	1.0 × 10^−2^	2.9	3281	87.6

In an attempt to represent the SS/electrolyte interface, the electrical equivalent circuit model is used to get insights about the double layer. A physical representation of the double-layer includes ohmic resistance of the electrolyte, *R*_s_, constant phase element, *Q*, and the charge transfer resistance, *R*_ct_. Because of heterogeneity of the electrode surface, the modelization of the double layer was approached using a constant phase element (CPE) instead of the double-layer capacitance (*C*_dl_).^58,59^ In this case, CPE matched precisely the impedance of the interface electrode/solution instead of the capacitor. The experimental results were fitted, and the simulated parameters according to the electrical equivalent circuit were gathered in Table 3.

**Table 3:** EIS parameters for the equivalent circuit for 316 steel in 0.5 M HCl solution in the presence of 1.0 × 10^−2^ M of C1, C2, and different concentrations of C3, at *T* = 291 K

Concentrations M		*R*_s_ (Ω cm^2^)	*R*_ct_ (Ω cm^2^)	*Q* (μΩ^−1^*S*^*n*^ cm^−2^)	*n*	*C*_dl_ (μF cm^−2^)	*τ* (ms)	IE_ct_ (%)
Blank		2.80	424	603	0.77	396.8	168.3	—
C1	1.0 × 10^−2^	2.74	1519	307	0.81	257.9	391.7	72.2
C2	1.0 × 10^−2^	1.90	2687	230	0.88	207.9	581.3	84.2
C3	1.0 × 10^−6^	1.70	1122	345	0.77	260.3	292.0	62.2
	1.0 × 10^−5^	1.37	1686	313	0.78	248.5	353.1	74.8
	1.0 × 10^−4^	3.22	2243	281	0.79	248.9	558.3	81.1
	1.0 × 10^−3^	2.12	2719	335	0.81	209.4	548.4	84.4
	1.0 × 10^−2^	3.34	3281	204	0.83	187.2	614.3	87.6

The double-layer capacitance values, *C*_dl_, for the electrical equivalent circuit including a CPE, is calculated using the eqn (9):^60,61^

9 *C*_dl_ = (*Q* × *R*_ct_^(1−*n*)^)^1/*n*^

where *R*_ct_ is the charge transfer resistance, *C*_dl_ is the double layer capacitance, *Q* is the CPE constant, and *n* is a CPE exponent.

The relaxation time (*τ*) is the time required to reach a steady-state after an electrical perturbation:^62^

10 *τ* = *C*_dl_ × *R*_cd_

where *τ* is the relaxation time, *R*_ct_ is the charge transfer resistance, and *C*_dl_ is the double-layer capacitance.

Close inspection of Table 3 shows that in the case of inhibited solutions, the CPE values are lower than those of the blank. This can be referred to the adsorption of the inhibitor substances on the metal surface to form a protective adsorption barrier against aggressive ions. The value of charge transfer resistance, *R*_ct_, increases while the double layer capacitance, *C*_dl_ of the interface that is considered as a capacitance, decreases after the addition of the inhibitors in acidic medium. The decrease in the capacity value could be due to the adsorption of the C1, C2, and C3 which protect the metal surface.^63^ The time constant values, *τ*, obtained by eqn (5) in the presence of C1, C2, and C3 were found to be higher than those found in the blank solution.
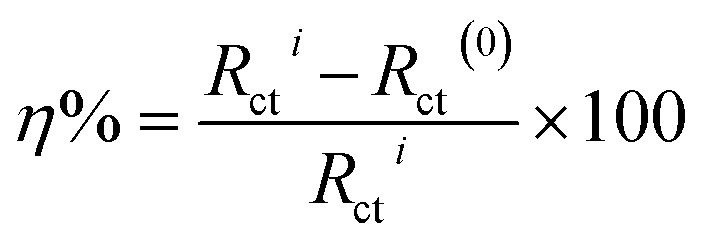


It was evident from Table 3, that the inhibitory efficiencies, IE_ct_ (%), computed from EIS parameters using eqn (9) were in accordance with the results obtained from polarization measurements.

The obtained results have shown that the best inhibition efficiency of the studied inhibitors tested have reached a value of 88% for a concentration of 1.0 × 10^−2^ M at *T* = 291 K. The inhibitory efficiencies of these studied inhibitors are linked to the presence of hydroxyl groups as active sites attached to the aromatic nucleus of these three derivatives. To increase the inhibition efficiency of these compounds, other derivatives of benzoic acids may be used in future studies, namely acids containing more than three hydroxyls groups^64–66^ or other radicals.^67–70^ Similar to multiple studies, it is scheduled to study the synergistic effect of iodide I^−^ ions on the inhibition efficiencies of the studied compounds.^35,71^ Likewise, these products can be used as corrosion inhibitors by their combination and their enhanced solidarity effect^72^ will be investigated.

### 3.2. Weight-loss measurements

**Fig. 4:**
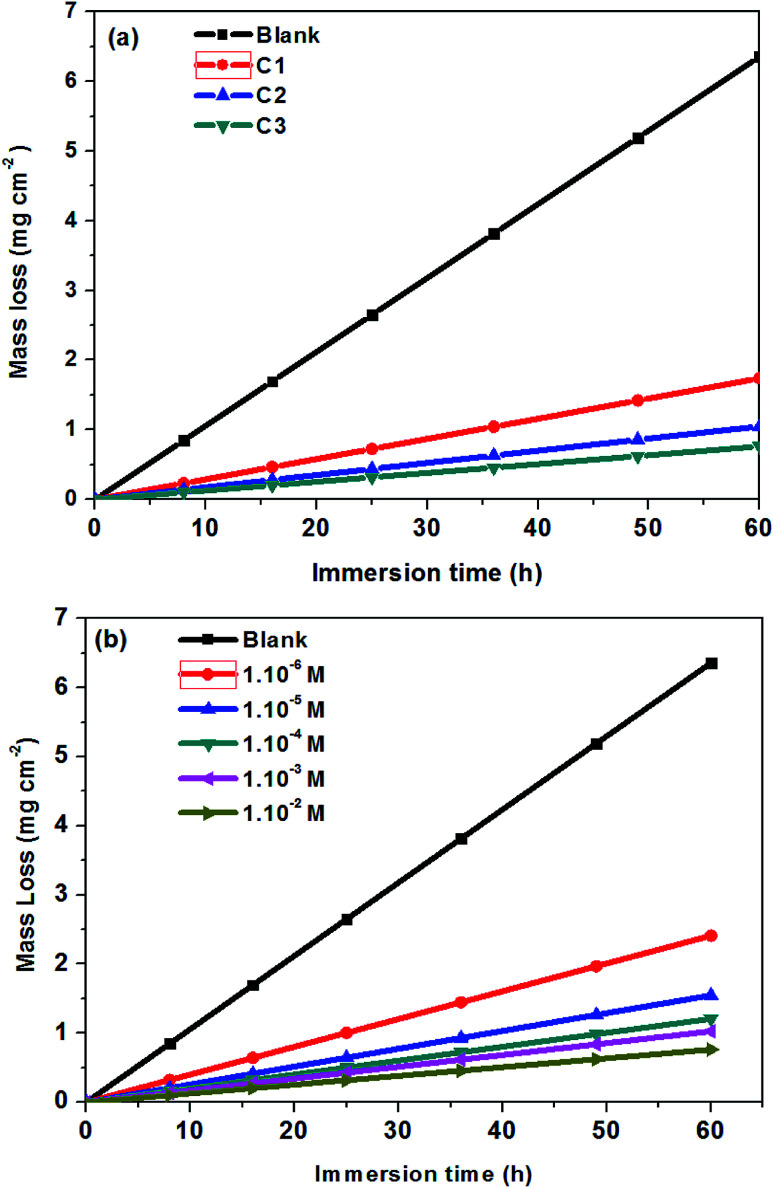
Mass loss of steel 316 *versus* immersion time at 291 K; (a) in 0.5 M HCl + 1.0 × 10^−2^ M of C1, C2, and C3; (b) in 0.5 M HCl solution at different concentrations of C3.

Weight-loss per surface unity (WL) of SS was determined at constant time intervals of up to 60 hours in 0.5 M HCl without and with 1.0 × 10^−2^ M inhibitor concentrations of C1, C2, and C3 at temperatures of 291 (±1) K. WL *versus* time of SS plates is linear with the correlation factors almost equal to unity (Fig. 4a) and Table 4. WL increased with time in the absence and the presence of the inhibitors. This behavior testifies that the corrosion products undergo dissolution in the aggressive medium.^29^ The slopes of the curves represent the corrosion rate (CR) given in mg cm^−2^ h^−1^. The CR of SS in the uninhibited solution was high compared to that of the inhibited acid solution. Besides, the CR in the presence of 1.0 × 10^−2^ M of C1 is greater than that in the presence of the same concentration of C2. The CRs were 0.029 mg cm^−2^ h^−1^, 0.017 mg cm^−2^ h^−1^ and 0.013 mg cm^−2^ h^−1^ for the inhibited solution containing 1.0 × 10^−2^ M C1, C2, and C3, respectively (Table 4). These results show that C3 inhibitor protects SS better than C1 and C2 in 0.5 mol L^−1^ HCl medium. Fig. 4b shows the WL of SS in the presence of various concentrations of C3 inhibitor. The CR decreased with increasing the inhibitor concentrations from 0.040 mg cm^−2^ h^−1^ to 0.013 mg cm^−2^ h^−1^ in the range of the used inhibitor concentrations (Table 4). The degree of metal surface coverage, *θ*, and the inhibition efficiency, IE (%), of the examined compounds were computed using eqn (1) and (2), respectively (Table 4). The inhibition efficiencies were 72.6%, 83.9%, and 88.0% for the solution containing 1.0 × 10^−2^ M of C1, C2, and C3, respectively. Close examination of the results indicates that C3 inhibitor is the most SS protective compared to C1 and C2 at the same concentrations. Variation of C3 inhibitor concentration in the aggressive medium from 1.0 × 10^−6^ M to 1.0 × 10^−2^ M leads to an increase of IE (%) from 62.3% to 88.0%.

**Table 4:** Gravimetric data and inhibition efficiency for 316 SS in 0.5 M HCl solution, 60 hours immersion time, *T* = 291 K

Concentration of the inhibitor in 0.5 M HCl (M)		CR (mg cm^−2^ h^−1^)	Correlation coefficient, *R*^2^	*θ*	IE (%)
0		0.11	0.998	—	—
C1	1.0 × 10^−6^	0.06	0.991	0.44	44.3
	1.0 × 10^−5^	0.05	0.987	0.58	57.6
	1.0 × 10^−4^	0.04	0.990	0.63	63.2
	1.0 × 10^−3^	0.03	0.986	0.69	68.9
	1.0 × 10^−2^	0.03	0.983	0.73	72.6
C2	1.0 × 10^−6^	0.04	0.990	0.58	58.5
	1.0 × 10^−5^	0.03	0.997	0.70	69.7
	1.0 × 10^−4^	0.03	0.998	0.72	72.4
	1.0 × 10^−3^	0.03	0.996	0.76	76.4
	1.0 × 10^−2^	0.02	0.996	0.84	83.9
C3	1.0 × 10^−6^	0.04	0.993	0.62	62.3
	1.0 × 10^−5^	0.03	0.992	0.76	75.6
	1.0 × 10^−4^	0.02	0.993	0.81	81.1
	1.0 × 10^−3^	0.02	0.996	0.84	83.9
	1.0 × 10^−2^	0.01	0.990	0.88	88.0

### 3.3. Adsorption studies

The surface coverage, *θ*, and the percent inhibition efficiency, IE%, are calculated from the gravimetric measurements using eqn (1) and (2), respectively and are showed in Table 4. The data were examined by fitting to several adsorption isotherms, including Langmuir, Villamil (modified Langmuir), El-Awady, Temkin, and Freundlich.

#### 3.3.1. Langmuir adsorption isotherm

According to the Langmuir isotherm, the surface coverage *θ* is related to the inhibitor concentration using the equation below:^73^11
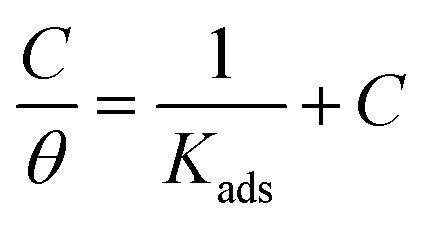
where, *C* is the concentration, *θ* is the surface coverage, and *K*_ads_ is the equilibrium constant of the adsorption process expressed in L mol^−1^. This constant is correlated to the standard Gibbs free energy of adsorption Δ*G*^0^_ads_ and calculated using the following equation:^74^12
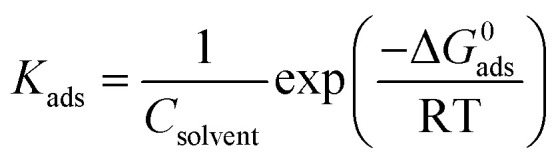
where *C*_solvent_ is the water molar concentration (*C* = 55.5 mol L^−1^), *T* is the temperature expressed in *K*, *R* is the gas constant, and Δ*G*^0^_ads_ is standard Gibbs free energy of adsorption.

**Fig. 5:**
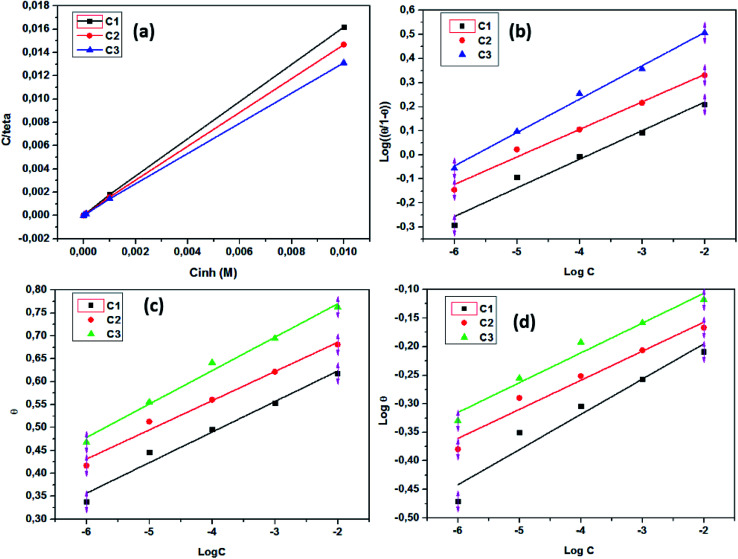
(a) Langmuir, (b) El-Awady, (c) Temkin, and (d) Freundlich isotherm models for stainless steel in 0.5 M HCl containing C1, C2 and C3 inhibitors at 308 K.

Fig. 5a shows the Langmuir adsorption isotherms plotted for 316 SS in inhibited 0.5 M HCl, at 308 K. The reverse of the *y*-axis intercept, in this figure, yielded *K*_ads_ in (L mol^−1^), and Δ*G*^0^_ads_ in (kJ mol^−1^) was then calculated using eqn (12). The standard Gibbs free energy, Δ*G*^0^_ads_, of adsorption of C1, C2, and C3 on the SS surface were determined and illustrated in Table 5, at 308 K.

**Table 5:** Adsorption parameters on 316 SS corrosion inhibition by C1, C2, and C3 compounds obtained from Langmuir, Villamil, El-Awady, Temkin, and Freundlich adsorption isotherms

Isotherm		*R* ^2^	Slope	IIntercept	*K*_ads_	Δ*G*^0^_ads_ (kJ mol^−1^)
Villamil (modified Langmuir)	C1	0.999	1.61	5.71 × 10^−5^	17 513.13	−35.33
	C2	0.999	1.46	4.30 × 10^−5^	23 255.81	−36.05
	C3	0.999	1.31	3.84 × 10^−5^	26 041.67	−36.34
El-Awady	C1	0.976	0.12	0.456	7318.24	−33.09
	C2	0.988	0.11	0.562	85 079.43	−39.38
	C3	0.995	0.14	0.785	487 984.24	−43.85
Temkin	C1	0.979	0.07, *a* = −17.45	0.757	6026.56	−32.05
	C2	0.986	0.06, *a* = −18.28	0.813	11 473.73	−33.67
	C3	0.990	0.07, *a* = −15.99	0.915	37 071.52	−36.63
Freundlich	C1	0.966	0.05	−0.055	0.881	−9.97
	C2	0.947	0.06	−0.071	0.849	−9.87
	C3	0.973	0.05	−0.002	0.995	−10.28

It can be seen that the correlation coefficients were almost unity for the inhibitors. However, the slope derived from unity as predicted by the eqn (11).

#### 3.3.2. Villamil adsorption isotherm

The correlation coefficients *R*^2^ values of Langmuir isotherm are 0.999; however, the slope of the curve derived from the unity. To try overcome this discrepancy, Shaban *et al.*^75,76^ reported that the deviation from the unity of the slope for the Langmuir isotherm could be due to the interactions between the adsorbed species, which adsorb on several sites, onto the metal surface and/or variation of adsorption heat with increasing surface coverage. These factors are not taken into account by Langmuir model. For that, the conventional Langmuir isotherm (eqn (14)) is modified and named Villamil isotherm: eqn (13).13
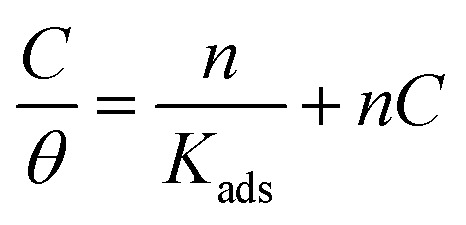
where *n* is the correction factor deduced from the slopes and referred to the number of displacements of adsorbed water (H_2_O) molecules from the metal surface.^77^Table 5 shows the equilibrium constant, the free energy of adsorption, the correlation coefficients the slopes, and the intercepts of the curves obtained for the studied inhibitors. The negative values of Δ*G*^0^_ads_ infer that the inhibitor adsorption onto SS surface is spontaneous. On the other hand, the absolute values of Δ*G*^0^_ads_ are less than −40 kJ mol^−1^ and up to −20 kJ mol^−1^, which indicates that the adsorption process on metal surface is a mixed adsorption.^78^

#### 3.3.3. El-Awady adsorption isotherm

According to El-Awady isotherm, *θ* is connected to the inhibitor concentration using eqn (14):^79^14
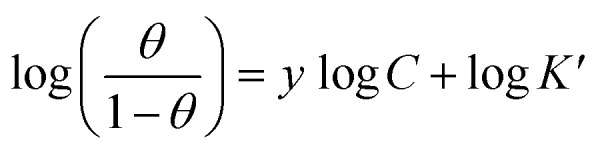
where *K*′ is a constant (*K*_ads_ = (*K*′)1/*y*), and *y* is the number of inhibitor molecules adsorbed one active site. Eqn (14) shows a straight line with slope and intercept of log *K*′, as presented in Fig. 5b. The values of 1/*y* are less than one, which implies an adsorption in multilayer forms, while the value of 1/*y* is >1 means that the inhibitor occupies several active sites.^79^ It is known that *K*_ads_ represents the strength between the adsorbate and the adsorbent. Then, large values of *K*_ads_ suggest better inhibition efficiency.^80^ In this study, *K*_ads_ value increased proportionally with the inhibition efficiency, indicating that the adsorption of C1, C2, and C3 on the SS surface was favorable. Δ*G*^0^_ads_ is found and its negative values indicate that the adsorption process is spontaneous. Its value, which is less than −40 kJ mol^−1^ indicates the occurrance of physisorption and chemisorption on the SS interface.^78^ The correlation factors are almost close to unity indicating a strong adherence to El-Awady adsorption isotherm.^79^ The adsorption parameters *i.e.*, *K*_ads_, the slope, the intercept, and the free energy are listed in Table 5.

#### 3.3.4. Temkin adsorption isotherm

The degree of surface coverage (*θ*) is connected to the inhibitor concentration (*C*) and the adsorption equilibrium constant *K*_ads_ as shown in eqn (15) and (16).^81,82^

15 exp(−2*a* *θ*) = *K*_ads_ × *C*

16
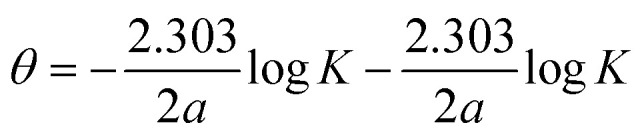
where *a* is the attractive parameter and *K* is the adsorption equilibrium constant.

From Fig. 5c linear plots are obtained, which affirms that the adsorption obeys the Temkin adsorption isotherm. Adsorption parameters obtained from this figure are shown in Table 5. The negative values of the attractive parameter, *a*, pointed out that there is a repulsion in the adsorption layer.

#### 3.3.5. Freundlich adsorption isotherm

According to the Freundlich isotherm, *θ* is related to the inhibitor concentration *C* by means of eqn (17):^83^

17 log *θ* = log *K*_ads_ + *n*log *C*

where *n* is the empirical constant, and the other constants have the same meaning.

Fig. 5d shows straight lines relation of log *θ* against log *C* with slope *n* and intercept log *K*_ads_. The deduced adsorption parameters *K*_ads_, *n*, and Δ*G*^0^_ads_ are shown in Table 5. The obtained values of the correlation factor are far from unity.

The inhibition efficiencies of C3 compound are superior compared to those of C2 and C1 for the same concentrations. The results obtained by gravimetric measurements, electrochemical techniques, and surface analysis are consistent. 3,4-Dihydroxybenzoic acid is a better inhibitor compared to benzoic acid and *para*-hydroxybenzoic acid; this can be explained by the mesomeric effect M which makes the previous compounds less active than 3,4-dihydroxybenzoic acid. The parallel correlation in corrosion protection of our electrode with the increase of benzoic acid concentrations may be explained by the basis of inhibitor adsorption in 0.5 M HCl medium. The adsorption process was studied using Langmuir, Villamil (modified Langmuir), El-Awady, Freundlich, and Temkin isotherms. The adsorption studies clearly indicated that the experimental data satisfied the Langmuir and Villamil adsorption isotherms with good linearity. The chosen criteria of the best fit isotherm are based on the higher correlation coefficient, *R*^2^. Villamil isotherm was more suitable as Langmuir isotherm was discarded because of the obtained intercepts which differed from unity (Table 5). Villamil isotherm led to the values of Δ*G*^0^_ads_ which were −35.33, −36.05, and −36.34 kJ mol^−1^ for C1, C2 and C3, respectively. According to these data, the adsorption is spontaneous and the inhibitors behave as mixed adsorption onto the metal surface as Δ*G*^0^_ads_ are less than −40 kJ mol^−1^ but up to −20 kJ mol^−1^.

### 3.4. Surface morphology studies

**Fig. 6:**
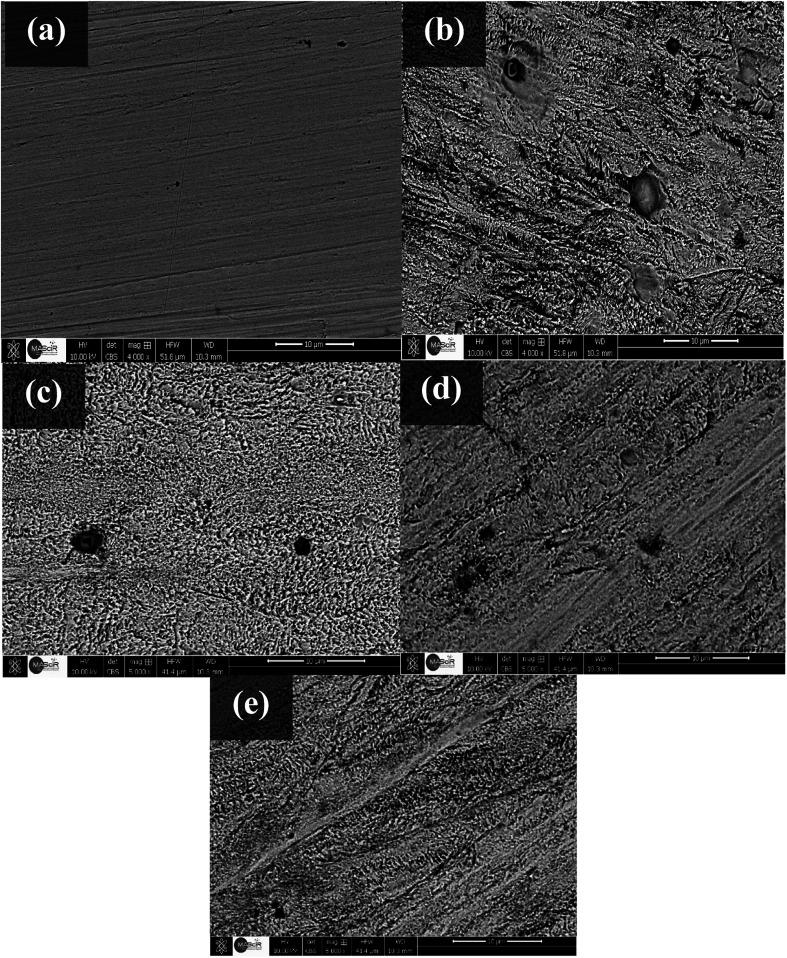
SEM micrographs of the surface of stainless steel; (a) before immersion; (b) after immersion in 0.5 M HCl; (c) after immersion in 0.5 M HCl + 1.0 × 10^−2^ M C1; (d) after immersion in 0.5 M HCl + 1.0 × 10^−2^ M C2; and (e) after immersion in 0.5 M HCl + 1.0 × 10^−2^ M C3. Immersion time = 48 hours, at 291 K.

In order to analyze the SS surface, samples were dipped in acidic media without and with the inhibitors, for 48 hours, at 291 K, (Fig. 6a–e). As it can be seen from (Fig. 6a), SEM image presents the polished surface of the SS prepared as showed before (Fig. 6b) presents the morphology of steel surface dipped in an uninhibited medium. Obvious surface roughness and pits are apparent in the absence of inhibitor. Fig. 6c–e show micrographs of the samples dipped in 0.5 M HCl containing the inhibitor C1, C2, and C3, respectively. These micrographs showed less surface roughness and pits compared to the steel surface immersed in uninhibited medium. The surface of steel dipped in acid solution containing C3 inhibitor showed relatively smoother surface and less pitted morphology than the steel dipped in the corrosive medium containing C1 and C2 inhibitors. This difference is as a result of the protective film of C3 molecule, which serves more than C1 and C2 as a protective layer, which hinted corrosive acid ions to approach the surface steel. The results are consistent with the other results carried out using electrochemical techniques and weight-loss measurements.

### 3.5.5 Computational investigation

In order to get further insights into the donor–acceptor interactions between the studied benzoic acid derivatives and the AISI 316 SS in an acidic environment and to explain and confirm the experimental results, quantum study was carried out. The first objective is the explanation of the reactivity of each compound (C1) *para*-hydroxybenzoic acid, (C2) benzoic acid, and (C3) 3,4-dihydroxybenzoic acid, respectively. Their adsorption behaviors at the SS in acid medium will be explained using Monte Carlo simulation. To confirm the inhibition efficiency of these three benzoic derivatives, the determined experimental results showed the following order: C3 > C2 > C1.

#### 3.5.1. Optimized structures and local reactivity parameters

**Fig. 7:**
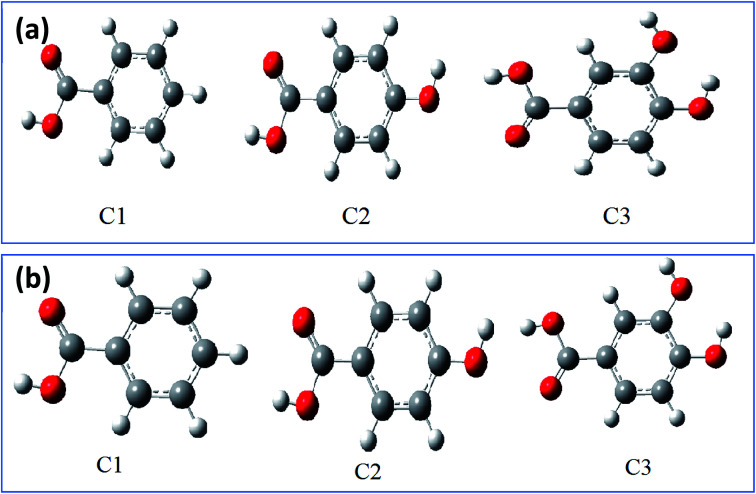
The optimized structures of inhibitor molecules in gas phase (a) and aqueous phase (b) using DFT/B3LYP/6-31G++(2d,p).

**Fig. 8:**
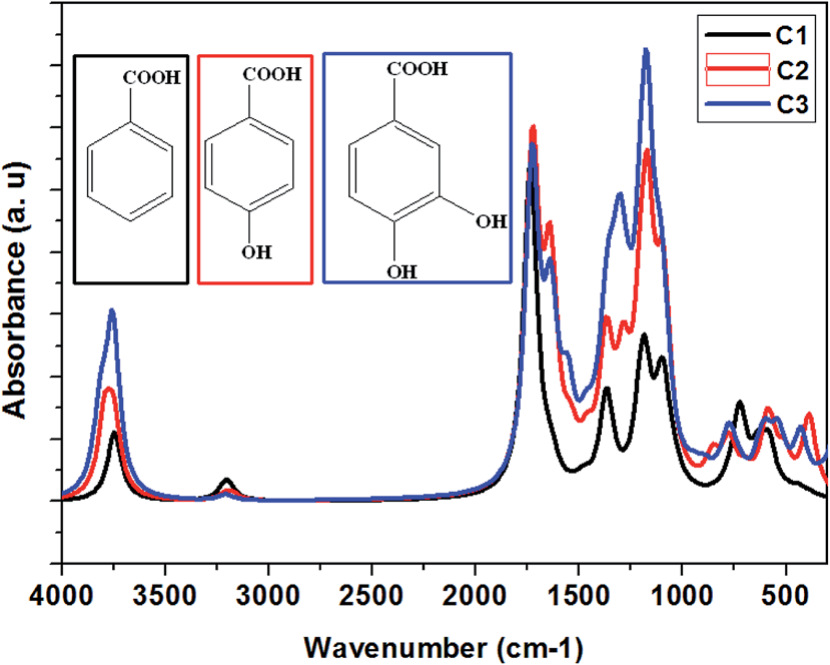
Theoretical vibrational spectra of C1, C2, and C3 inhibitors.

To corroborate the experimental results, the corrosion inhibition efficiencies of the three compounds (C3, C2, and C1) were studied in this part using quantum chemical calculations. The latter is conditioned by the determination of the optimal structure which allows to study molecules at the minima of their potential energy.^84,85^ Thus, the optimized structure of studied inhibitors C1, C2, and C3 in aqueous and gas phases have been determined as shown in the Fig. 7 and 8 using density functional calculation at B3LYP/6-31G++(2d,p) basis set,^86^ to subsequently determine the other local and global parameters.

The benzoic acid derivatives: (C3) 3,4-dihydroxybenzoic acid, (C2) *para*-hydroxybenzoic acid, and (C1) benzoic acid contain several functional groups such as hydroxyl and carboxylic groups (Table 1). In order to shed more light on the active sites of studied molecules, the vibrational spectroscopy of all molecules was calculated using the B3LYP/6-31G++(2d,p) method (Fig. 8), which aims to identify the functional groups in C1, C2, and C3 as a potential hub for their interactions with the studied steel surface. The vibrational spectra of C1, C2, and C3 have primarily confirmed the existence of carboxylic acid and alcohol groups. As shown in Fig. 8 the peaks at around 1731, 1727, and 1720 cm^−1^ are attributed to carboxyl groups in C1, C2, and C3, respectively. Moreover, and in the same order, the peaks at 3745, 3775, and 3759 cm^−1^ are attributed to hydroxyl groups.

**Fig. 9:**
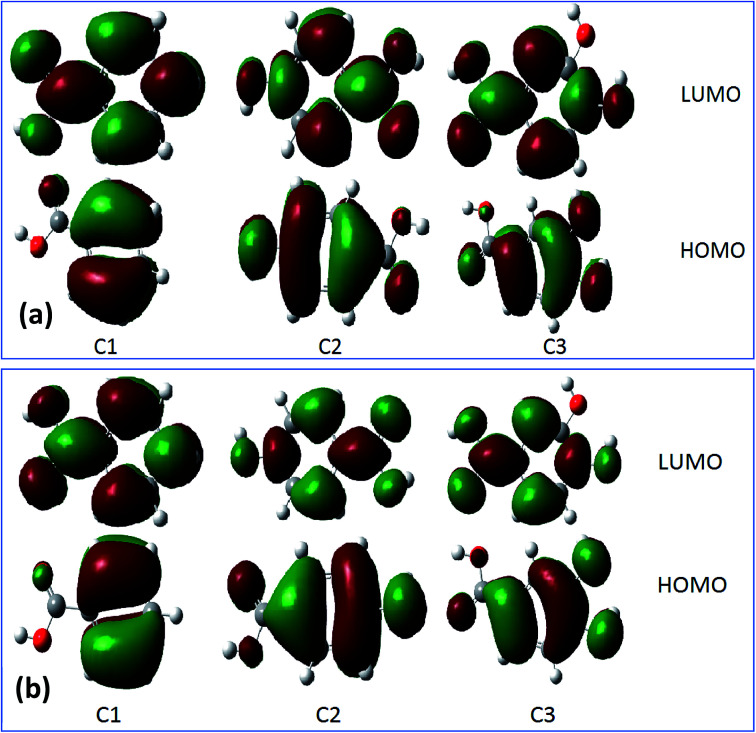
The HOMOs and LUMOs of inhibitor molecules in gas phase (a) and aqueous phase (b) using DFT/B3LYP/6-31G++(2d,p).

**Fig. 10:**
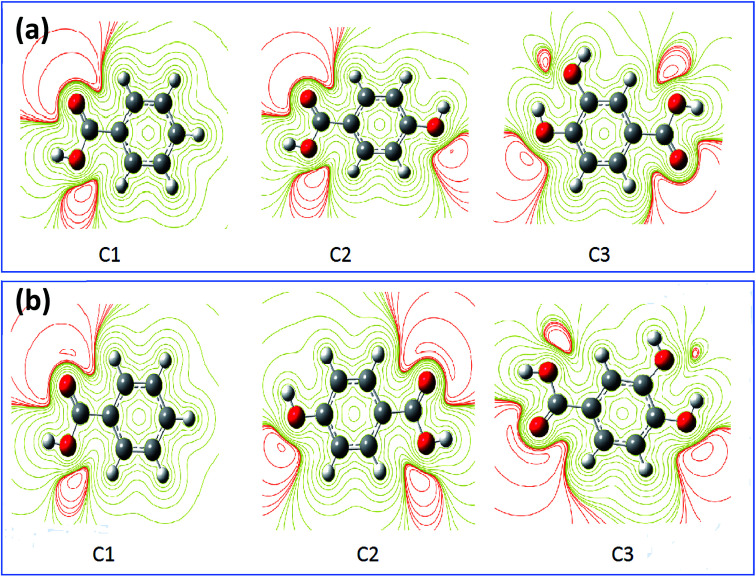
The contour and isosurface representation of electrostatic potential regions of negative (positive) potential are red (green) of molecules in gas phase (a) and aqueous phase (b) using DFT/B3LYP/6-31G++(2d,p).

For more information about the reactivity of the tested inhibitors, some local descriptors of reactivity were determined for these molecules in gas and aqueous phase such as the density distributions of highest occupied molecular orbital (HOMO) and lowest unoccupied molecular orbital (LUMO) that are shown in Fig. 9a and b. The electrostatic potential maps (ESP) exhibited in Fig. 10a and b show the reactive regions distribution of the studied inhibitors which indicate the most interacting sites.^87^ More precisely, the density distributions of HOMOs in the inhibitor compound indicate the sites that have the highest tendency to donate electrons to the electron-poor system,^88^ such as the metal surface targeted for protection, which is often positively charged in a corrosive environment.^61^ Moreover, the LUMOs distribution gives an indication on the regions with the highest capability to accept electrons from a donor electron of a potential reactive.^62^ After analyzing the results shown in Fig. 9 of inhibitor molecules in gas and aqueous phase using DFT/B3LYP/6-31G++(2d,p), the calculated results of HOMOs noted that its regions centered remarkably around carboxylic function (COOH) and from the side connected of phenyl with carboxylic function. Thus, it can be said that the tested inhibitors C1, C2, and C3 giving up preferably its p electron density through its (COOH) functional groups, as a result to facilitate their adsorption over the metallic surface.

The results obtained from HOMOs and LUMOs are consistent with the maps ESP inferences as illustrated in Fig. 10. While considering, in the particular emplacement of highest occupied molecular orbitals as a region of electronic density is important, it is noted that it matches with the red regions. So, it is evident that the areas of congruence identify the active centers or the active areas in each compound.^89^ In addition, the contour maps of electron density reveals that the oxygen atoms exhibited as favorable interaction sites,^90^ notably those are part of the acid function in comparison with the other atoms. These favorable oxygen atoms are surrounded by dark red color contours (Fig. 10). This distinction between the oxygen atoms in the hydroxyl function (OH) and the carboxylic function (COOH) was noticed in the case of (C3) 3,4-dihydroxybenzoic acid, and (C2) *para*-hydroxybenzoic acid. Therefore, the oxygen atoms of the carboxylic function was preferably a more active site in C3 and C2 as adsorption sites to form bonding between metal surface and inhibitors.^91^ Then, a good assessment of local reactivity parameters such as HOMOs, LUMOs and ESP maps are related to oxygen atoms, which greatly confirms the interaction of the corrosion inhibitor C3, C2 and C1 with the metal surface considered locally through the oxygen atoms in this case, existing in functional groups COOH and OH, respectively.

#### 3.5.2. Mulliken charge distribution and global reactivity parameters

All the theoretical quantum calculations were performed with deprotonated forms of the studied molecules using fundamentally Lee–Yang–Parr correlation functional (B3LYP) and the Pople-type 6-31G++(d,p), and 6-31G++(d,p) basis set.^92^ In order to confirm the validity of the results obtained by these two methods, we used other methods, namely Hartree Fock (HF), and Møller–Plesset perturbation theory method (MP2), with the same bases 6-31G+(d,p) and 6-31G++(d,p).^85,93^ The calculated quantum chemical parameters in the aqueous and gas phase are presented in Tables A1 and A2,† respectively. Relevant quantum chemical parameters with the studied benzoic acid derivatives were derived based on electronic properties of their optimized structures. Frontier molecular orbitals energies, that is the highest occupied molecular orbital energy (EHOMO), the lowest unoccupied molecular orbital energy (ELUMO), and the energy gap (Δ*E* = ELUMO − EHOMO) were calculated for all the inhibitors. Then, all the other quantum parameters which globally describes the inhibitors reactivity such as chemical hardness (*η*), softness (*σ*), electronegativity (*χ*), electrophilicity (*ω*), nucleophilicity (*ε*), and particularly the fraction of transferred electrons Δ*N* were calculated.

According to the obtained results for benzoic acid molecules by these various methods and basis sets, the values of HOMO energy of studied molecules suggested that the highest values are linked in this order to C3, C2, and C1, respectively. This clue of high-energy value of HOMO states shows that the C3 compound is prone to donate electrons to appropriate acceptor metal surface more than C2, and C1, respectively. In the same context, the results of the energy gap (Δ*E*, eqn (3)) express that C3 has a low energy gap, which shows that this inhibitor can efficiently react and easily adsorbs onto the metal surface compared to C2 and C1, respectively.^94^ Applying the eqn (4)–(6) above that based on first vertical ionization energy and electron affinity values of the inhibitor compounds allowed us to determine the other quantum chemical parameters summarized in Tables A1 and A2.† Chemical hardness and softness are quantum chemical parameters closely associated with each other as shown by Koopman's theorem.^95^ Knowing that the hardness is defined as the resistance towards the electron cloud polarization and deformation of chemical species, and global softness can be defined as the inverse of the global hardness. Therefore, the calculated chemical hardness and softness can confirm the corrosion inhibition efficiency ranking of the studied compounds as following C3 > C2 > C1, which fully matches the experimental results. According to Pearson, the fraction of electrons transferred (Δ*N*) is determined by the (eqn (6)).^96^ This parameter describes the reactivity of the studied inhibitors globally as a combination of calculated chemical hardness and the electronegativity, as this factor is a quantification of electrons transferred from the inhibitor to the metallic surface. Knowing that, the electron transferred from the inhibitor to the metallic surface increases when the electronegativity of the inhibitor decreases.^97^ According to the obtained results of (Δ*N*), C3 has the great values followed by C2, and C1. The experimental values of the corrosion inhibition efficiencies of inhibitors have the same order, which shows that the experimental and theoretical results are in good agreement.

**Fig. 11:**
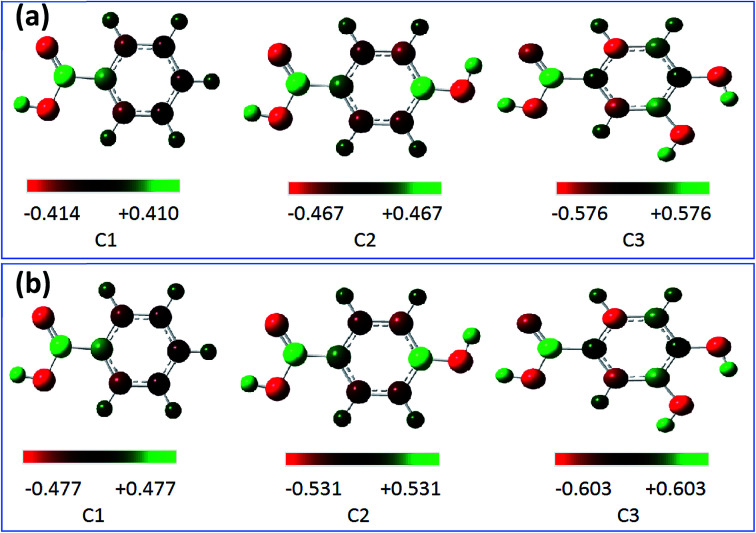
Mulliken charge distribution for the molecules calculated at the B3LYP/6-31G++(2d,p) in gas phase (a) and aqueous phase (b).

The Mulliken charges of all atoms (C and O) were illustrated in Table 3A.† The charges of O11, O12, O14, and O16 atoms were chosen for discussion as the most favorable sites for the interaction with the metal surface and are shown in Table 6. These atoms as active centers with excess charges could act as a nucleophilic reagent.^98^ Generally, it can be shown from the Mulliken charge distribution calculated at the B3LYP/6-31G++(2d,p) and the location of each active site inside the studied compounds *via*Fig. 11a and b, that the best sites that can act as an electron donor.

**Table 6:** The calculated Mulliken charges (in a.u.) of the oxygen atom (O) as active sites for C1, C2 and C3 molecules calculated at the B3LYP/6-31G++(2d,p) in gas and aqueous phases

Inhibitors	Phases	Oxygen atoms (O)			
		O11	O12	O14	O16
C1	Gas	−0.378627	−0.408691		
	Aq.	−0.409812	−0.411073		
C2	Gas	−0.379705	−0.410034	−0.515089	
	Aq.	−0.410723	−0.412260	−0.536031	
C3	Gas	−0.348481	−0.444866	−0.524060	−0.531439
	Aq.	−0.407392	−0.453940	−0.569537	−0.575114

#### 3.5.3. Monte Carlo simulation

**Fig. 12:**
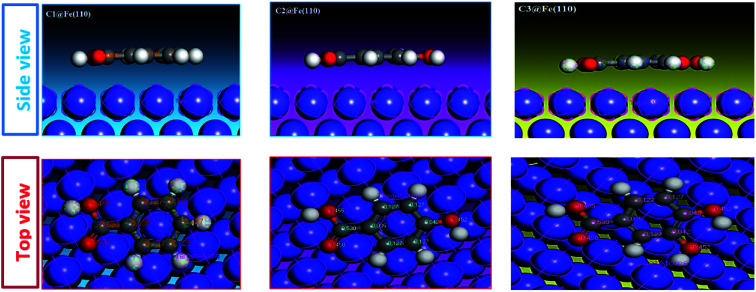
Molecular simulation for the adsorption mode of carboxylic acid derivatives on iron surface (110).

The adsorption behaviors of C1, C2, and C3 derivatives were conducted using Monte Carlo simulations on the Fe (110) surface. To perceive the stable adsorption configuration (energy and structure) of the studied molecules on the metal surface, Fig. 12. Shows how C1, C2, and C3 could be adsorbed on the Fe (110) surface *via* two different views. From the side view, all inhibitor molecules are found to be parallel on the surface. From the top view, all molecules are flat on the metal surface.

The interaction energy and the binding energy of the three inhibitors on the surface of iron (110) are listed in Table 7. The examination of this table shows that the obtained adsorption energy increases in the order: C1 ˃ C2 ˃ C3, also the binding energies (positive value) calculated for the interactions between inhibitors and the metal surface are high. It is important to note that high binding energy leads to a more stable inhibitor–surface interaction, which indicates that the adsorption of the inhibitor on the metal surface is easier and the inhibition efficiency is high.

**Table 7:** The adsorption and binding energies of carboxylic acid derivatives on iron surface (110) (all units in (kcal mol^−1^))

Inhibitor	*E*_T_	*E*_inh_	*E*_ads_	*E*_bind_
C1	−80.9549	−9.5900	−71.3649	71.3649
C2	−101.2423	−25.25267	−75.9896	75.9896
C3	−102.6706	−23.25017	−79.4204	79.4204

C3 has the highest binding energy (*E*_bind_) compared to the other compounds (*E*_bind_ (C3) = 79.4204, *E*_bind_ (C2) = 75.9896 and *E*_bind_ (C1) = 71.3649). This confirms the efficiency of C3 as significant inhibition. The behavior of the C3 molecule is mainly due to the presence of the benzene ring and the four oxygen atoms in its molecular structure. There is a good correlation between the experimental inhibition efficiencies of the previous results and the binding energies calculated in this study.

## 4. Conclusion

The inhibition efficiency of benzoic acid (C1), *para*-hydroxybenzoic acid (C2), and 3,4-dihydroxybenzoic acid (C3) towards reducing the corrosion rate (or enhancing the corrosion resistance) of austenitic AISI 316 stainless steel (SS) have been evaluated in 0.5 M HCl using weight loss (WL), open circuit potential (OCP), potentiodynamic polarization method, electrochemical impedance spectroscopy (EIS), and scanning electron microscopy (SEM) analysis. The different experimental techniques were in good agreement, showing that C3 (3,4-dihydroxybenzoic acid) is a better inhibitor compared to C1 (benzoic acid) and C2 (*para*-hydroxybenzoic acid) at equal concentrations, and the inhibition efficiencies increased with the increase of concentration of the inhibitors. Thus, the adsorption of these compounds onto the SS surface from the aqueous medium are well fitted to Villamil adsorption isotherm for the three inhibitors. In addition, the obtained Gibbs free energy Δ*G*^0^_ads_, for the inhibitor adsorption, is negative, which implies the spontaneity of adsorption and the stability of the adsorbed layer on steel surfaces. Δ*G*^0^_ads_ values were laying between −40 and −20 kJ mol^−1^ ensuring the mixed adsorption process. At the same time the theoretical calculations of C1, C2, and C3 inhibitor molecules in the gas and aqueous phases which were considered in DFT/B3LYP/6-31G++(2d,p) and Monte Carlo simulations, showed that the electronic and global reactivity parameters of these three benzoic acid derivatives are in good agreement with the experimentally determined inhibition efficiencies. Therefore, the inhibition capabilities of the studied inhibitors that adsorb on a parallel orientation are ranked in the following order: C3 > C2 > C1. It should be noted, that this original work is the first part of a vast work, which is aimed to improve the performance of new inhibitors and contribute to more protection of AISI 316 stainless steel in hydrochloric acid medium. The achieved inhibition efficiency of even 88% pushes us to carry out further research to overcome this limitation and to improve the inhibition efficiency based on the synergistic effect by adding another compound (organic compounds, or mineral ions) to the inhibited medium in order to maximize the inhibition efficiency.

## Conflicts of interest

There are no conflicts to declare.

## Supplementary Material

RA-010-D0RA06742C-s001
