# Supplementary material for: Experimental and theoretical investigations of benzoic acid derivatives as corrosion inhibitors for AISI 316 stainless steel in hydrochloric acid medium: DFT and Monte Carlo simulations on the Fe (110) surface
Source: RSC Adv. 2020 Nov 12;10(67):41137–53. doi: 10.1039/d0ra06742c (PMC9057760; doi:10.1039/d0ra06742c)
Supplement: RA-010-D0RA06742C-s001 [file RA-010-D0RA06742C-s001.pdf]

This journal is © The Royal Society of Chemistry 2020

Supporting information

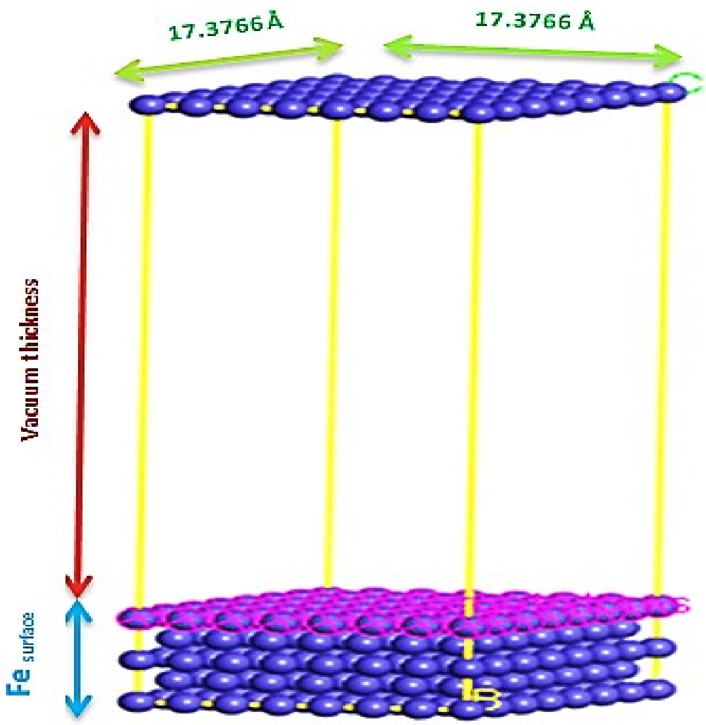

Figure 1A. Simulation box adopted in the Monte Carlo study.

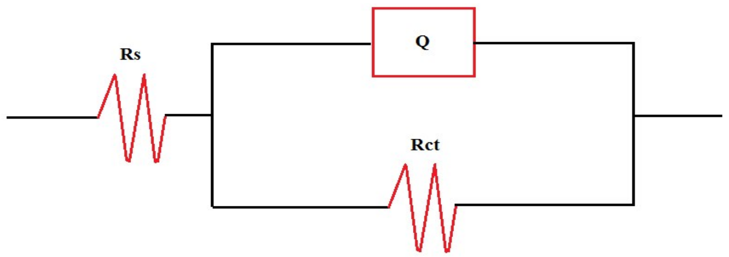

Figure 2A. Electrical equivalent circuit of the double layer model.

Table A1. Calculated quantum chemical parameters for the molecules in gaz phase (eV)

gaz	ELUMO	EHOMO	ELUMO	EHOMO(ev)	ΔE	Energy	Energie	I	A	η	χ	ω	ε	ΔN	
HF/6-31G+(2d,p)															
C1	0,04231	-0,34992	1,15131433	-9,52181309	10,6731274	-256,778786	-6964,45694	9,52181309	-1,15131433	5,33656371	4,18524938	1,64116024	0,60932502	0,02761614	72,7
C2	0,04292	-0,33323	1,16791329	-9,06765482	10,2355681	-311,827036	-8457,4976	9,06765482	-1,16791329	5,11778406	3,94987077	1,52424164	0,65606396	0,05179285	84,1
C3	0,0434	-0,32255	1,18097476	-8,77703707	9,95801183	-486,356087	-13191,1443	8,77703707	-1,18097476	4,97900592	3,79803116	1,44858641	0,69032816	0,06848444	88,2
HF/6-31G++(2d,p)															
C1	0,07062	-0,34993	1,92166907	-9,5220852	11,4437543	-256,778856	-6964,45885	9,5220852	-1,92166907	5,72187714	3,80020807	1,26196185	0,79241698	0,05940288	72,7
C2	0,06992	-0,33324	1,90262109	-9,06792694	10,970548	-311,827126	-8457,50003	9,06792694	-1,90262109	5,48527401	3,58265292	1,16998731	0,85471012	0,08179601	84,1
C3	0,06721	-0,32256	1,82887819	-8,77730918	10,6061874	-486,356154	-13191,1462	8,77730918	-1,82887819	5,30309369	3,4742155	1,13803131	0,87871045	0,09482998	88,2
MP_2/6-31G+(2d,p)															
C1	0,09993	-0,324236	2,7192352	-8,82291549	11,5421507	-257,623677	-6987,37242	8,82291549	-2,7192352	5,77107535	3,05184014	0,80693179	1,23926213	0,12373429	72,7
C2	0,11239	-0,32351	3,05828925	-8,80316001	11,8614493	-312,850508	-8485,25662	8,80316001	-3,05828925	5,93072463	2,87243538	0,69560514	1,4375972	0,13552852	84,1
C3	0,10816	-0,31241	2,94318502	-	11,4442985	-	-	8,50111347	-	5,72214925	2,77896423	0,67480258	1,4819149	0,14863609	88,2

				8,50111347		489,326547	13271,7103		2,94318502						
MP_2/6-31G++(2d,p)															
C1	0,06423	-0,27182	1,74778822	-7,39660275	9,14439097	-257,623841	-6987,37685	7,39660275	-1,74778822	4,57219549	2,82440726	0,87236825	1,14630489	0,18105008	72,7
C2	0,05471	-0,24906	1,48873569	-6,77727128	8,26600698	-312,850801	-8485,26457	6,77727128	-1,48873569	4,13300349	2,6442678	0,84589236	1,18218351	0,2220821	84,1
C3	0,05612	-0,243738	1,52710377	-6,63245221	8,15955598	-488,031289	-13236,5798	6,63245221	-1,52710377	4,07977799	2,55267422	0,79859072	1,25220589	0,23620474	88,2
B3LYP/6-31G+(2d,p)															
C1	0,06422	-0,26182	1,74751611	-7,12448875	8,87200486	-258,272896	-7004,98079	7,12448875	-1,74751611	4,43600243	2,68848632	0,81469282	1,2274565	0,20192884	72,7
C2	0,05471	-0,24806	1,48873569	-6,75005988	8,23879558	-313,64174	-8506,71674	6,75005988	-1,48873569	4,11939779	2,6306621	0,83997509	1,19051149	0,22446702	84,1
C3	0,05611	-0,23738	1,52683165	-6,45944213	7,98627379	-489,353282	-13272,4354	6,45944213	-1,52683165	3,99313689	2,46630524	0,76163949	1,31295712	0,25214447	88,2
B3LYP/6-31G++(2d,p)							0								
C1	0,04644	-0,24992	1,26369742	-6,80067309	8,0643705	-258,272976	-7004,98296	6,80067309	-1,26369742	4,03218525	2,76848784	0,95041825	1,05216835	0,21223134	72,7
C2	0,03585	-0,23432	0,97552869	-6,37617525	7,35170394	-313,641872	-8506,7203	6,37617525	-0,97552869	3,67585197	2,70032328	0,99184432	1,00822274	0,24207677	84,1
C3	0,03653	-0,22105	0,99403244	-6,01507997	7,00911241	-489,353382	-13272,4382	6,01507997	-0,99403244	3,50455621	2,51052376	0,89921936	1,1120757	0,28098797	88,2

Table A2. Calculated quantum chemical parameters for the molecules in aqueous phase (eV)

aqu	**E**LUMO	**E**HOMO	**E**LUMO	**E**HOMO(ev)	ΔE	**E**nergy	Energie	**I**	**A**	η	χ	ω	ε	ΔN	
HF/6-31G+(2d,p)															
**C1**	0,032806	-0,3064941	0,89269719	-8,34013355	9,23283074	-256,778786	-6964,45694	8,34013355	-0,89269719	4,61641537	3,72371818	1,50182295	0,66585745	0,08191224	72,7
**C2**	0,03249	-0,302194	0,88409839	-8,22312181	9,1072202	-311,827036	-8457,4976	8,22312181	-0,88409839	4,5536101	3,66951171	1,47853197	0,67634655	0,08899404	84,1
**C3**	0,03491	-0,303246	0,94994997	-8,2517482	9,20169818	-486,356087	-13191,1443	8,2517482	-0,94994997	4,60084909	3,65089912	1,44854396	0,69034839	0,09010303	88,2

HF/6-31G++(2d,p)															
**C1**	0,05996	-0,313245	1,92166907	-9,5220852	11,4437543	-256,778856	-6964,45885	9,5220852	-1,92166907	5,72187714	3,80020807	1,26196185	0,79241698	0,05940288	72,7
**C2**	0,05472	-0,302194	1,90262109	-9,06792694	10,970548	-311,827126	-8457,50003	9,06792694	-1,90262109	5,48527401	3,58265292	1,16998731	0,85471012	0,08179601	84,1
**C3**	0,06681	-0,3054942	1,82887819	-8,77730918	10,6061874	-486,356154	-13191,1462	8,77730918	-1,82887819	5,30309369	3,4742155	1,13803131	0,87871045	0,09482998	88,2
MP_2/6-31G+(2d,p)															
**C1**	0,10402	-0,31402	2,83052983	-8,54492383	11,3754537	-257,623677	-6987,37242	8,54492383	-2,83052983	5,68772683	2,857197	0,71764828	1,39344026	0,14265831	72,7
**C2**	0,179605	-0,34409	4,8873035	-9,36317063	14,2504741	-312,850508	-8485,25662	9,36317063	-4,8873035	7,12523706	2,23793356	0,35145123	2,84534501	0,15733276	84,1
**C3**	0,1763	-0,3252	4,79736982	-8,84914728	13,6465171	-489,326547	-13271,7103	8,84914728	-4,79736982	6,82325855	2,02588873	0,30075257	3,32499232	0,17983426	88,2
MP_2/6-31G++(2d,p)															
**C1**	0,06673	-0,2557129	1,81581672	-6,95830601	8,77412273	-257,623841	-6987,37685	6,95830601	-1,81581672	4,38706136	2,57124464	0,75349972	1,32714051	0,21754373	72,7
**C2**	0,05827	-0,24845	1,58560828	-6,76067233	8,34628061	-312,850801	-8485,26457	6,76067233	-1,58560828	4,1731403	2,58753203	0,80219229	1,2465839	0,22674387	84,1
**C3**	0,05923	-0,23633	1,61173122	-6,43087016	8,04260138	-488,031289	-13236,5798	6,43087016	-1,61173122	4,02130069	2,40956947	0,72190884	1,38521645	0,25743294	88,2
B3LYP/6-31G+(2d,p)															
**C1**	0,04975	-0,246125	1,35376715	-6,69740583	8,05117298	-258,272896	-7004,98079	6,69740583	-1,35376715	4,02558649	2,67181934	0,88665572	1,12783347	0,22458599	72,7
**C2**	0,04024	-0,23513	1,09498674	-6,39821648	7,49320322	-313,64174	-8506,71674	6,39821648	-1,09498674	3,74660161	2,65161487	0,9383252	1,06572859	0,24400581	84,1
**C3**	0,04081	-0,22151	1,11049723	-6,02759721	7,13809445	-489,353282	-13272,4354	6,02759721	-1,11049723	3,56904722	2,45854999	0,84679015	1,18093013	0,28319183	88,2
B3LYP/6-31G++(2d,p)							0								
**C1**	0,059826	-0,2436844	1,62794922	-6,63099368	8,2589429	-258,272976	-7004,98296	6,63099368	-1,62794922	4,12947145	2,50152223	0,75767729	1,31982311	0,23955581	72,7
**C2**	0,0592	-0,23632	1,61091488	-6,43059805	8,04151293	-313,641872	-8506,7203	6,43059805	-1,61091488	4,02075646	2,40984158	0,72216964	1,38471621	0,25743395	84,1
**C3**	0,6671	-0,27128	18,1527249	-7,38190859	25,5346335	-489,353382	-13272,4382	7,38190859	-18,1527249	12,7673168	-5,38540817	1,13581506	0,88042503	0,38635401	88,2

Table 3A. Mulliken charge distribution for the molecules calculated at the B3LYP/6-31G++(2d, p) in gas and aqueous phases.

	Atom	C1	C2	C3	C4	C5	C6	C10	O11	O12	O14	O16
C1	gas	-0.09110	-0.085454	-0.173841	0.174220	-0.171249	-0.081242	0.406797	-0.378627	-0.408691		
	aq	-0.10093	-0.097685	-0.182301	0.174314	-0.181060	-0.093672	0.408368	-0.409812	-0.411073		
C2	gas	0.37222	-0.169179	-0.167000	0.155045	-0.164094	-0.165285	0.411120	-0.379705	-0.410034	-0.515089	
	aq	0.378340	-0.178138	-0.175422	0.156103	-0.173661	-0.174062	0.412745	-0.410723	-0.412260	-0.536031	
C3	gas	-0.107261	0.192333	-0.149921	0.291486	-0.723088	0.336202	0.429496	-0.348481	-0.444866	-0.524060	-0.531439
	aq	-0.056133	0.201193	-0.157283	0.321261	-0.739296	0.287849	0.469892	-0.407392	-0.453940	-0.569537	-0.575114
